# Supplementary material for: Comparative assessment of macrophage responses and antileishmanial efficacy in dynamic vs. Static culture systems utilizing chitosan-based formulations
Source: PLoS One. 2025 Mar 11;20(3):e0319610. doi: 10.1371/journal.pone.0319610 (PMC11896045; doi:10.1371/journal.pone.0319610)
Supplement: S1 Table — (The data presented in this table were used to generate Fig 2). Values behind the means, standard deviations. (DOCX) [file pone.0319610.s001.docx]

**S1 Table: Phagocytosis of fluorescent latex beads (2 μm) by uninfected and infected PEMs, BMMs, and THP-1 in static culture system. (The data presented in this table were used to generate Figure 2).** **Values behind the means, standard deviations.**

***Number of latex beads *10^5^/mg protein**

| Time/Hour | Uninfected PEMs | Uninfected BMMs | Uninfected THP-1 | Infected PEMs | Infected BMMs | Infected THP-1 |
| --- | --- | --- | --- | --- | --- | --- |
| 0.5 | 2.14, 2.59, 2.53 | 2.03, 2.51, 2.36 | 1.21, 0.73, 1.06 | 3.45, 3.50, 3.40 | 2.94, 3.03, 3.03 | 1.80, 1.85, 1.75 |
| 1 | 6.38, 8.06, 6.35 | 7.32, 5.80, 5.48 | 5.81, 5.72, 4.07 | 11.55, 11.59, 11.55 | 10.93, 10.89, 10.89 | 7.97, 8.01, 8.02 |
| 2 | 61.18, 63.02, 59.34 | 60.38, 60.99, 58.63 | 42.27, 40.91, 39.82 | 76.58, 77.07, 76.09 | 73.86, 74.28, 73.86 | 59.00, 59.24, 58.75 |
| 4 | 117.27, 103.86, 99.08 | 89.33, 101.49, 94.17 | 60.18, 72.39, 65.43 | 143.45, 137.96, 147.47 | 139.28, 138.84, 138.88 | 92.83, 88.59, 88.59 |
| 24 | 442.88, 444.20, 376.73 | 433.58, 383.05, 371.37 | 227.67, 276.70, 290.63 | 496.62, 574.76, 518.62 | 484.96, 527.74, 544.30 | 368.61, 403.84, 421.55 |

*Phagocytosis was significantly higher (p<0.05 by t-test) in infected macrophages compared to uninfected ones. Initial macrophage infection rate was >80% after 24 h*, n=1*.*
