# Supplementary material for: Comparative assessment of macrophage responses and antileishmanial efficacy in dynamic vs. Static culture systems utilizing chitosan-based formulations
Source: PLoS One. 2025 Mar 11;20(3):e0319610. doi: 10.1371/journal.pone.0319610 (PMC11896045; doi:10.1371/journal.pone.0319610)
Supplement: S2 Table — (DOCX) [file pone.0319610.s002.docx]

**S2 Table: Phagocytosis of fluorescent latex beads (2 μm) by uninfected and infected PEMs, BMMs, and THP-1 in static culture system.**

***Number of latex beads *10^5^/mg protein**

| Time/Hour | Uninfected PEMs | Uninfected BMMs | Uninfected THP-1 | Infected PEMs | Infected BMMs | Infected THP-1 |
| --- | --- | --- | --- | --- | --- | --- |
| 0.5 | 1.92, 2.36, 2.32 | 1.82, 2.28, 2.20 | 1.06, 0.86, 1.08 | 3.38, 3.42, 3.25 | 2.93, 3.04, 3.04 | 1.80, 1.85, 1.75 |
| 1 | 5.63, 7.09, 5.58 | 6.65, 5.95, 5.70 | 5.61, 5.53, 3.87 | 11.49, 11.53, 11.49 | 10.83, 10.79, 10.79 | 7.96, 8.01, 8.03 |
| 2 | 60.57, 61.69, 59.24 | 59.54, 59.86, 57.60 | 41.05, 40.30, 38.65 | 76.42, 76.72, 75.76 | 73.97, 74.38, 73.65 | 59.02, 59.48, 58.50 |
| 4 | 108.19, 101.87, 95.94 | 85.29, 93.41, 97.29 | 57.74, 63.82, 67.44 | 142.10, 135.77, 145.13 | 139.82, 135.37, 135.81 | 93.83, 89.59, 89.59 |
| 24 | 460.82, 416.53, 396.14 | 418.56, 377.79, 367.65 | 223.68, 268.09, 279.24 | 490.87, 564.90, 513.23 | 481.13, 523.12, 537.75 | 368.61, 403.84, 421.55 |

*Phagocytosis was significantly higher (p<0.05 by t-test) in infected macrophages compared to uninfected ones. Initial macrophage infection rate was >80% after 24 h, n=2.*
