# Supplementary material for: Comparative assessment of macrophage responses and antileishmanial efficacy in dynamic vs. Static culture systems utilizing chitosan-based formulations
Source: PLoS One. 2025 Mar 11;20(3):e0319610. doi: 10.1371/journal.pone.0319610 (PMC11896045; doi:10.1371/journal.pone.0319610)
Supplement: S3 Table — (DOCX) [file pone.0319610.s003.docx]

**S3 Table: Phagocytosis of fluorescent latex beads (2 μm) by uninfected and infected PEMs, BMMs, and THP-1 in static culture system.**

***Number of latex beads *10^5^/mg protein**

| Time/Hour | Uninfected PEMs | Uninfected BMMs | Uninfected THP-1 | Infected PEMs | Infected BMMs | Infected THP-1 |
| --- | --- | --- | --- | --- | --- | --- |
| 0.5 | 1.82, 2.25, 2.23 | 1.72, 2.17, 2.11 | 0.90, 0.97, 1.13 | 3.32, 3.34, 3.24 | 2.93, 3.04, 3.04 | 1.85, 1.79, 1.76 |
| 1 | 5.53, 6.99, 5.48 | 6.44, 5.78, 5.48 | 5.65, 5.59, 4.35 | 11.49, 11.53, 11.49 | 10.83, 10.79, 10.79 | 8.00, 8.04, 7.96 |
| 2 | 61.20, 62.85, 60.45 | 59.54, 59.86, 57.60 | 42.47, 39.96, 37.57 | 76.18, 76.37, 75.45 | 73.87, 74.79, 73.34 | 58.35, 58.21, 57.44 |
| 4 | 110.27, 104.15, 100.58 | 87.27, 97.06, 91.67 | 60.06, 64.62, 67.33 | 140.87, 135.97, 143.16 | 140.82, 136.42, 136.76 | 92.83, 88.59, 88.59 |
| 24 | 456.02, 422.53, 405.46 | 416.75, 379.89, 370.37 | 229.88, 268.53, 278.59 | 504.08, 560.81, 519.10 | 489.00, 526.86, 541.14 | 371.84, 401.42, 417.74 |

*Phagocytosis was significantly higher (p<0.05 by t-test) in infected macrophages compared to uninfected ones. Initial macrophage infection rate was >80% after 24 h, n=3.*
