# Supplementary material for: Comparative assessment of macrophage responses and antileishmanial efficacy in dynamic vs. Static culture systems utilizing chitosan-based formulations
Source: PLoS One. 2025 Mar 11;20(3):e0319610. doi: 10.1371/journal.pone.0319610 (PMC11896045; doi:10.1371/journal.pone.0319610)
Supplement: S4 Table — Values behind the means, standard deviations. (DOCX) [file pone.0319610.s004.docx]

| **S4 Table: Phagocytosis of fluorescent latex beads (2 μm) by infected PEMs, BMMs and THP-1 in the three culture systems (static, slow flow rate 1.45 x 10⁻⁹ m/s and fast flow rate 1.23 x 10^-7^ m/s ) (The data presented in this table were used to generate Figure 3). Values behind the means, standard deviations.** | | | | | | | | | |
| --- | --- | --- | --- | --- | --- | --- | --- | --- | --- |
|  | **Number of latex beads *10^5^/mg protein** | | | | | | | | |
|  | **infected cells - static system** | | | **Infected cells - 1.45 x 10^-9^ m/s** | | | **Infected cells - 1.23 x 10^-7^ m/s** | | |
| **Time/Hour** | **PEMs** | **BMMs** | **THP-1** | **PEMs** | **BMMs** | **THP-1** | **PEMs** | **BMMs** | **THP-1** |
| 0.5 | 3.50, 3.40, 3.44 | 3.04, 2.95, 3.01 | 1.83, 1.74, 1.82 | 1.09, 1.06, 1.04 | 1.02, 1.01, 0.97 | 1.01, 1.01, 0.97 | 0.65, 0.57, 0.41 | 0.57, 0.45, 0.33 | 0.28, 0.43, 0.19 |
| 1 | 11.56, 11.58, 11.53 | 10.92, 10.87, 10.91 | 8.00, 8.03, 7.98 | 6.47, 6.58, 6.72 | 5.98, 5.89, 6.13 | 3.02, 3.24, 3.04 | 3.99, 3.93, 3.84 | 3.97, 3.84, 3.86 | 1.43, 1.57, 1.50 |
| 2 | 77.14, 76.32, 76.27 | 74.20, 74.23, 73.58 | 59.26, 58.97, 58.77 | 40.81, 39.96, 39.96 | 39.85, 39.31, 39.34 | 22.20, 22.48, 22.81 | 28.25, 28.38, 27.91 | 26.72, 27.18, 27.10 | 14.94, 14.79, 15.27 |
| 4 | 142.17, 138.63, 148.08 | 134.90, 140.10, 142.00 | 89.37, 89.66, 93.97 | 72.48, 71.60, 83.68 | 68.23, 73.47, 80.90 | 51.11, 50.39, 48.50 | 50.51, 49.68, 60.46 | 52.84, 52.82, 44.34 | 36.23, 33.77, 29.00 |
| 24 | 506.35, 507.22, 576.57 | 539.96, 540.47, 476.57 | 415.66, 416.86, 367.48 | 289.53, 279.75, 342.36 | 286.51, 284.16, 338.33 | 223.62, 222.80, 159.58 | 252.12, 188.68, 252.52 | 245.07, 245.49, 192.25 | 161.84, 150.81, 119.35 |
| Flow conditions caused a significant reduction in phagocytosis by infected macrophages (p>0.05 by one-way ANOVA). *Initial macrophage infection rate was >80% after 24 h, n=1.* | | | | | | | | | |
