# Supplementary material for: Comparative assessment of macrophage responses and antileishmanial efficacy in dynamic vs. Static culture systems utilizing chitosan-based formulations
Source: PLoS One. 2025 Mar 11;20(3):e0319610. doi: 10.1371/journal.pone.0319610 (PMC11896045; doi:10.1371/journal.pone.0319610)
Supplement: S5 Table — (DOCX) [file pone.0319610.s005.docx]

| **S5 Table: Phagocytosis of fluorescent latex beads (2 μm) by infected PEMs, BMMs and THP-1 in the three culture systems (static, slow flow rate 1.45 x 10⁻⁹ m/s and fast flow rate 1.23 x 10^-7^ m/s ).** | | | | | | | | | |
| --- | --- | --- | --- | --- | --- | --- | --- | --- | --- |
|  | **Number of latex beads *10^5^/mg protein** | | | | | | | | |
|  | **infected cells - static system** | | | **Infected cells - 1.45 x 10^-9^ m/s** | | | **Infected cells - 1.23 x 10^-7^ m/s** | | |
| **Time/Hour** | **PEMs** | **BMMs** | **THP-1** | **PEMs** | **BMMs** | **THP-1** | **PEMs** | **BMMs** | **THP-1** |
| 0.5 | 3.01, 3.04, 2.94 | 2.96, 3.05, 2.98 | 1.74, 1.82, 1.84 | 1.07, 1.08, 1.03 | 0.97, 1.02, 1.01 | 0.97, 1.02, 1.01 | 0.67, 0.52, 0.43 | 0.57, 0.46, 0.32 | 0.44, 0.20, 0.26 |
| 1 | 11.56, 11.58, 11.53 | 10.90, 10.92, 10.88 | 7.98, 8.03, 7.99 | 6.73, 6.50, 6.54 | 6.02, 5.90, 5.78 | 3.14, 2.95, 2.91 | 3.99, 3.84, 3.92 | 3.96, 3.90, 3.81 | 1.50, 1.57, 1.43 |
| 2 | 76.37, 77.14, 76.23 | 74.42, 73.83, 73.74 | 859.19, 859.09, 858.72 | 40.28, 40.71, 39.73 | 38.81, 38.84, 39.35 | 21.66, 22.10, 22.24 | 28.05, 28.03, 28.46 | 27.01, 26.75, 27.24 | 15.12, 14.72, 15.17 |
| 4 | 147.03, 144.15, 137.70 | 136.88, 143.24, 136.88 | 87.71, 92.59, 89.70 | 71.79, 72.28, 83.69 | 76.92, 77.93, 66.85 | 49.76, 49.66, 47.59 | 59.93, 52.70, 48.02 | 46.89, 47.46, 55.65 | 34.44, 35.74, 28.82 |
| 24 | 506.79, 576.58, 506.79 | 497.61, 497.96, 561.43 | 413.20, 413.91, 366.89 | 287.88, 281.18, 342.58 | 282.59, 283.27, 333.84 | 236.03, 162.58, 201.39 | 189.65, 244.05, 259.63 | 241.89, 192.45, 248.46 | 156.37, 157.08, 118.55 |
| Flow conditions caused a significant reduction in phagocytosis by infected macrophages (p>0.05 by one-way ANOVA). *Initial macrophage infection rate was >80% after 24 h, n=2.* | | | | | | | | | |
