# Supplementary material for: Comparative assessment of macrophage responses and antileishmanial efficacy in dynamic vs. Static culture systems utilizing chitosan-based formulations
Source: PLoS One. 2025 Mar 11;20(3):e0319610. doi: 10.1371/journal.pone.0319610 (PMC11896045; doi:10.1371/journal.pone.0319610)
Supplement: S6 table — (DOCX) [file pone.0319610.s006.docx]

| **S6 table: Phagocytosis of fluorescent latex beads (2 μm) by infected PEMs, BMMs and THP-1 in the three culture systems (static, slow flow rate 1.45 x 10⁻⁹ m/s and fast flow rate 1.23 x 10^-7^ m/s ).** | | | | | | | | | |
| --- | --- | --- | --- | --- | --- | --- | --- | --- | --- |
|  | **Number of latex beads *10^5^/mg protein** | | | | | | | | |
|  | **infected cells - static system** | | | **Infected cells - 1.45 x 10^-9^ m/s** | | | **Infected cells - 1.23 x 10^-7^ m/s** | | |
| **Time/Hour** | **PEMs** | **BMMs** | **THP-1** | **PEMs** | **BMMs** | **THP-1** | **PEMs** | **BMMs** | **THP-1** |
| 0.5 | 3.44, 3.54, 3.46 | 3.10, 3.05, 3.00 | 1.87, 1.78, 1.81 | 1.09, 1.10, 1.05 | 1.02, 1.02, 0.98 | 0.97, 1.01, 1.02 | 0.69, 0.48, 0.47 | 0.55, 0.51, 0.32 | 0.42, 0.33, 0.18 |
| 1 | 11.77, 11.75, 11.72 | 11.11, 11.11, 11.07 | 8.08, 8.13, 8.10 | 6.64, 6.84, 6.62 | 5.98, 5.89, 6.13 | 3.02, 3.24, 3.04 | 3.98, 4.08, 3.94 | 3.96, 3.90, 3.81 | 1.52, 1.64, 1.65 |
| 2 | 76.72, 76.93, 77.65 | 74.92, 74.33, 74.24 | 59.66, 59.62, 59.22 | 40.50, 40.34, 41.26 | 39.85, 39.31, 39.34 | 22.20, 22.48, 22.81 | 28.63, 28.65, 28.22 | 27.01, 26.75, 27.24 | 15.26, 15.08, 15.56 |
| 4 | 143.65, 139.16, 149.19 | 143.33, 140.80, 135.87 | 89.37, 89.66, 93.97 | 70.29, 74.10, 83.61 | 68.23, 73.47, 80.90 | 51.11, 50.39, 48.50 | 57.51, 57.70, 46.79 | 46.89, 47.46, 55.65 | 36.40, 35.56, 29.44 |
| 24 | 511.31, 582.38, 511.31 | 545.59, 543.52, 479.88 | 415.66, 416.86, 367.48 | 302.86, 273.38, 341.75 | 286.51, 284.16, 338.33 | 223.62, 222.80, 159.58 | 258.18, 252.84, 190.98 | 241.89, 192.45, 248.46 | 149.88, 121.90, 166.22 |
| Flow conditions caused a significant reduction in phagocytosis by infected macrophages (p>0.05 by one-way ANOVA). *Initial macrophage infection rate was >80% after 24 h, n=3*. | | | | | | | | | |
