# Supplementary material for: Comparative assessment of macrophage responses and antileishmanial efficacy in dynamic vs. Static culture systems utilizing chitosan-based formulations
Source: PLoS One. 2025 Mar 11;20(3):e0319610. doi: 10.1371/journal.pone.0319610 (PMC11896045; doi:10.1371/journal.pone.0319610)
Supplement: S7 table — (The data presented in this table were used to generate Fig 4). Values behind the means, standard deviations. (DOCX) [file pone.0319610.s007.docx]

**S7 table: Macropinocytosis of pHrodo™ Red dextran by uninfected and infected PEMs, BMMs and THP-1 in static culture system. (The data presented in this table were used to generate Figure 4). Values behind the means, standard deviations.**

* **Concentration of dextran µg/mg protein**

| Time/Hour | Uninfected PEMs | Uninfected BMMs | Uninfected THP-1 | Infected PEMs | Infected BMMs | Infected THP-1 |
| --- | --- | --- | --- | --- | --- | --- |
| 0.5 | 0.44, 0.42, 0.43 | 0.31, 0.30, 0.29 | 0.16, 0.14, 0.14 | 1.06, 0.85, 0.85 | 0.70, 0.63, 0.46 | 0.39, 0.34, 0.16 |
| 1 | 1.70, 1.07, 1.07 | 1.11, 1.09, 1.09 | 0.51, 0.57, 0.57 | 2.79, 3.05, 2.56 | 2.08, 2.33, 2.19 | 1.46, 1.68, 1.66 |
| 2 | 3.48, 2.42, 2.42 | 3.09, 1.86, 2.55 | 1.06, 0.95, 0.95 | 3.97, 4.27, 3.09 | 3.52, 3.69, 2.99 | 1.84, 1.78, 1.78 |
| 4 | 6.04, 3.88, 4.57 | 3.97, 4.16, 4.16 | 2.63, 2.44, 2.44 | 7.89, 7.40, 6.00 | 6.08, 6.65, 4.97 | 4.32, 4.18, 3.2 |
| 24 | 20.47, 18.78, 17.81 | 16.67, 17.76, 15.08 | 9.56, 7.22, 7.22 | 24.35, 26.51, 25.04 | 22.19, 24.09, 22.71 | 14.53, 13.38, 12.59 |

*Macropinocytosis was significantly higher (p<0.05 by t-test) in infected macrophages compared to uninfected ones. Initial macrophage infection rate was >80% after 24 h, n=1.*
