# Supplementary material for: Comparative assessment of macrophage responses and antileishmanial efficacy in dynamic vs. Static culture systems utilizing chitosan-based formulations
Source: PLoS One. 2025 Mar 11;20(3):e0319610. doi: 10.1371/journal.pone.0319610 (PMC11896045; doi:10.1371/journal.pone.0319610)
Supplement: S8 Table — (DOCX) [file pone.0319610.s008.docx]

**S8 Table: Macropinocytosis of pHrodo™ Red dextran by uninfected and infected PEMs, BMMs and THP-1 in static culture system.**

* **Concentration of dextran µg/mg protein**

| Time/Hour | Uninfected PEMs | Uninfected BMMs | Uninfected THP-1 | Infected PEMs | Infected BMMs | Infected THP-1 |
| --- | --- | --- | --- | --- | --- | --- |
| 0.5 | 0.51, 0.47, 0.47 | 0.32, 0.37, 0.35 | 0.21, 0.22, 0.17 | 1.10, 1.07, 0.83 | 0.75, 0.69, 0.51 | 0.41, 0.45, 0.22 |
| 1 | 1.18, 1.67, 1.95 | 1.53, 1.42, 0.80 | 0.63, 0.69, 0.63 | 3.23, 3.43, 2.94 | 2.42, 2.64, 2.44 | 1.89, 1.88, 1.63 |
| 2 | 3.96, 3.15, 2.49 | 3.50, 3.12, 2.08 | 1.20, 1.33, 1.21 | 4.83, 4.73, 3.94 | 4.09, 4.00, 3.31 | 2.15, 2.21, 1.93 |
| 4 | 6.87, 4.42, 5.51 | 5.75, 5.23, 3.42 | 3.02, 2.93, 3.05 | 7.12, 8.34, 7.64 | 6.46, 6.43, 6.61 | 4.54, 4.34, 4.32 |
| 24 | 19.30, 21.85, 21.85 | 18.27, 19.32, 16.41 | 10.00, 9.68, 7.31 | 26.01, 28.37, 26.61 | 24.10, 26.23, 24.67 | 15.05, 15.76, 13.59 |

*Macropinocytosis was significantly higher (p<0.05 by t-test) in infected macrophages compared to uninfected ones. Initial macrophage infection rate was >80% after 24 h, n=2.*
