# Supplementary material for: Comparative assessment of macrophage responses and antileishmanial efficacy in dynamic vs. Static culture systems utilizing chitosan-based formulations
Source: PLoS One. 2025 Mar 11;20(3):e0319610. doi: 10.1371/journal.pone.0319610 (PMC11896045; doi:10.1371/journal.pone.0319610)
Supplement: S9 Table — (DOCX) [file pone.0319610.s009.docx]

**S9 Table: Macropinocytosis of pHrodo™ Red dextran by uninfected and infected PEMs, BMMs and THP-1 in static culture system.**

* **Concentration of dextran µg/mg protein**

| Time/Hour | Uninfected PEMs | Uninfected BMMs | Uninfected THP-1 | Infected PEMs | Infected BMMs | Infected THP-1 |
| --- | --- | --- | --- | --- | --- | --- |
| 0.5 | 0.44, 0.45, 0.46 | 0.31, 0.31, 0.33 | 0.16, 0.18, 0.17 | 1.02, 1.02, 0.81 | 0.70, 0.68, 0.48 | 0.40, 0.38, 0.18 |
| 1 | 1.57, 1.44, 1.05 | 1.11, 1.17, 1.17 | 0.56, 0.62, 0.56 | 3.01, 3.16, 2.68 | 2.21, 2.44, 2.25 | 1.71, 1.79, 1.55 |
| 2 | 2.91, 2.78, 2.86 | 2.54, 2.60, 2.66 | 1.01, 1.12, 1.02 | 4.00, 4.08, 3.62 | 3.68, 3.60, 3.22 | 1.96, 1.98, 1.76 |
| 4 | 4.93, 4.94, 5.13 | 4.34, 4.17, 4.38 | 2.55, 2.77, 2.64 | 7.03, 7.87, 7.01 | 5.90, 6.37, 6.03 | 4.48, 4.43, 3.39 |
| 24 | 19.02, 21.36, 19.03 | 17.31, 18.16, 15.52 | 9.25, 8.89, 6.76 | 25.09, 27.22, 25.69 | 22.91, 24.85, 23.64 | 14.10, 14.77, 12.84 |

*Macropinocytosis was significantly higher (p<0.05 by t-test) in infected macrophages compared to uninfected ones. Initial macrophage infection rate was >80% after 24 h, n=3.*
