# Supplementary material for: Comparative assessment of macrophage responses and antileishmanial efficacy in dynamic vs. Static culture systems utilizing chitosan-based formulations
Source: PLoS One. 2025 Mar 11;20(3):e0319610. doi: 10.1371/journal.pone.0319610 (PMC11896045; doi:10.1371/journal.pone.0319610)
Supplement: S10 Table — (The data presented in this table were used to generate Fig 5). Values behind the means, standard deviations. (DOCX) [file pone.0319610.s010.docx]

| **S10 Table: Macropinocytosis of pHrodo™ Red dextran by infected PEMs, BMMs and THP-1 at the three culture systems (static, slow flow rate 1.45 x 10⁻⁹ m/s and fast flow rate 1.23 x 10^⁻7^ m/s ). (The data presented in this table were used to generate Figure 5). Values behind the means, standard deviations.** | | | | | | | | | |
| --- | --- | --- | --- | --- | --- | --- | --- | --- | --- |
|  | **Concentration of dextran µg/mg protein** | | | | | | | | |
|  | **infected cells - static system** | | | **Infected cells -1.45 x 10^-9^ m/s** | | | **Infected cells - 1.23 x 10^-7^ m/s** | | |
| **Time/Hour** | **PEMs** | **BMMs** | **THP-1** | **PEMs** | **BMMs** | **THP-1** | **PEMs** | **BMMs** | **THP-1** |
| 0.5 | 1.04, 0.92, 0.80 | 0.73, 0.57, 0.49 | 0.31, 0.42, 0.17 | 0.30, 0.30, 0.28 | 0.21, 0.19, 0.20 | 0, 0, 0 | 0, 0, 0 | 0.17, 0.06, 0.07 | 0, 0, 0 |
| 1 | 2.76, 2.70, 2.94 | 2.06, 2.25, 2.29 | 1.54, 1.74, 1.52 | 0.74, 0.68, 0.62 | 0.61, 0.55, 0.49 | 0.32, 0.22, 0.21 | 0.21, 0.12, 0.06 | 1.12, 1.06, 1.12 | 0, 0, 0 |
| 2 | 3.65, 4.19, 3.49 | 3.08, 3.80, 3.31 | 1.67, 1.81, 1.92 | 1.68, 1.77, 1.80 | 1.56, 1.44, 1.51 | 0.76, 0.70, 0.64 | 1.16, 1.19, 1.60 | 1.99, 1.96, 2.05 | 0.48, 0.24, 0.34 |
| 4 | 7.67, 7.17, 6.46 | 6.43, 6.04, 5.23 | 3.63, 4.60, 3.46 | 3.52, 4.44, 3.29 | 2.59, 3.54, 2.87 | 1.50, 1.58, 1.41 | 2.39, 2.24, 2.24 | 9.95, 9.44, 7.62 | 1.02, 0.78, 0.90 |
| 24 | 26.38, 25.34, 24.18 | 22.64, 22.25, 24.11 | 14.45, 13.55, 12.49 | 14.88, 14.00, 16.42 | 15.38, 14.94, 14.65 | 9.03, 9.01, 8.96 | 10.62, 10.13, 7.87 | 9.95, 9.44, 7.62 | 3.22, 5.49, 3.56 |
| Flow conditions caused a significant reduction in macropinocytosis by infected macrophages (p>0.05 by one-way ANOVA). *Initial macrophage infection rate was >80% after 24 h, n=1*. | | | | | | | | | |
