# Supplementary material for: Comparative assessment of macrophage responses and antileishmanial efficacy in dynamic vs. Static culture systems utilizing chitosan-based formulations
Source: PLoS One. 2025 Mar 11;20(3):e0319610. doi: 10.1371/journal.pone.0319610 (PMC11896045; doi:10.1371/journal.pone.0319610)
Supplement: S11 Table — (DOCX) [file pone.0319610.s011.docx]

| **S11 Table: Macropinocytosis of pHrodo™ Red dextran by infected PEMs, BMMs and THP-1 at the three culture systems (static, slow flow rate 1.45 x 10⁻⁹ m/s and fast flow rate 1.23 x 10^⁻7^ m/s ).** | | | | | | | | | |
| --- | --- | --- | --- | --- | --- | --- | --- | --- | --- |
|  | **Concentration of dextran µg/mg protein** | | | | | | | | |
|  | **infected cells - static system** | | | **Infected cells -1.45 x 10^-9^ m/s** | | | **Infected cells - 1.23 x 10^-7^ m/s** | | |
| **Time/Hour** | **PEMs** | **BMMs** | **THP-1** | **PEMs** | **BMMs** | **THP-1** | **PEMs** | **BMMs** | **THP-1** |
| 0.5 | 1.13, 1.11, 0.91 | 0.81, 0.64, 0.57 | 0.36, 0.33, 0.31 | 0.33, 0.31, 0.35 | 0.23, 0.21, 0.18 | -, -, - | -, -, - | -, -, - | -, -, - |
| 1 | 2.63, 3.36, 3.01 | 1.99, 2.70, 2.51 | 1.87, 2.13, 1.40 | 1.03, 0.65, 0.57 | 0.65, 0.82, 0.34 | 0.41, 0.17, 0.26 | 0.24, 0.12, 0.07 | 0.19, 0.12, 0.05 | 0.04, 0.02, 0.02 |
| 2 | 3.78, 4.50, 4.01 | 3.46, 4.02, 3.32 | 2.26, 2.36, 1.68 | 1.92, 2.05, 1.58 | 1.68, 1.80, 1.33 | 0.73, 0.97, 0.85 | 1.80, 1.32, 1.08 | 1.42, 1.25, 0.93 | 0.68, 0.23, 0.29 |
| 4 | 8.30, 7.80, 6.41 | 6.52, 6.59, 5.49 | 4.51, 4.30, 3.79 | 2.86, 4.15, 2.90 | 2.66, 3.63, 3.01 | 2.07, 2.02, 1.31 | 1.86, 2.82, 2.52 | 1.66, 1.25, 0.69 | 1.59, 0.90, 0.50 |
| 24 | 27.18, 26.59, 24.83 | 24.89, 24.50, 22.60 | 15.09, 14.24, 12.67 | 17.13, 16.36, 14.51 | 15.18, 14.72, 15.70 | 10.50, 9.99, 9.52 | 11.27, 10.74, 8.29 | 8.23, 10.74, 8.64 | 6.15, 6.94, 4.31 |
| Flow conditions caused a significant reduction in macropinocytosis by infected macrophages (p>0.05 by one-way ANOVA). *Initial macrophage infection rate was >80% after 24 h, n=2.* | | | | | | | | | |
