# Supplementary material for: Comparative assessment of macrophage responses and antileishmanial efficacy in dynamic vs. Static culture systems utilizing chitosan-based formulations
Source: PLoS One. 2025 Mar 11;20(3):e0319610. doi: 10.1371/journal.pone.0319610 (PMC11896045; doi:10.1371/journal.pone.0319610)
Supplement: S12 Table — (DOCX) [file pone.0319610.s012.docx]

| **S12 Table: Macropinocytosis of pHrodo™ Red dextran by infected PEMs, BMMs and THP-1 at the three culture systems (static, slow flow rate 1.45 x 10⁻⁹ m/s and fast flow rate 1.23 x 10^⁻7^ m/s ).** | | | | | | | | | |
| --- | --- | --- | --- | --- | --- | --- | --- | --- | --- |
|  | **Concentration of dextran µg/mg protein** | | | | | | | | |
|  | **infected cells - static system** | | | **Infected cells -1.45 x 10^-9^ m/s** | | | **Infected cells - 1.23 x 10^-7^ m/s** | | |
| **Time/Hour** | **PEMs** | **BMMs** | **THP-1** | **PEMs** | **BMMs** | **THP-1** | **PEMs** | **BMMs** | **THP-1** |
| 0.5 | 0.91, 1.03, 1.21 | 0.86, 0.66, 0.57 | 0.49, 0.36, 0.20 | 0.36, 0.31, 0.32 | 0.25, 0.21, 0.21 | 0.00, 0.00, 0.00 | 0.00, 0.00, 0.00 | 0.00, 0.00, 0.00 | 0.00, 0.00, 0.00 |
| 1 | 3.41, 3.16, 2.73 | 2.31, 2.78, 2.11 | 1.44, 2.12, 1.69 | 1.00, 0.74, 0.51 | 0.86, 0.48, 0.40 | 0.44, 0.23, 0.23 | 0.26, 0.12, 0.07 | 0.20, 0.13, 0.03 | 0.09, 0.03, 0.03 |
| 2 | 4.81, 4.31, 3.48 | 4.07, 3.71, 2.87 | 2.37, 2.21, 1.72 | 1.63, 2.12, 1.80 | 1.75, 1.91, 1.43 | 1.19, 0.76, 0.46 | 1.87, 1.60, 1.03 | 1.61, 1.30, 1.00 | 0.72, 0.52, 0.12 |
| 4 | 8.80, 7.76, 6.24 | 7.37, 6.41, 4.82 | 3.23, 5.72, 3.95 | 4.18, 4.03, 1.99 | 3.94, 3.73, 1.93 | 2.44, 1.88, 1.22 | 2.35, 3.17, 1.97 | 2.46, 2.57, 1.57 | 1.74, 1.32, 0.54 |
| 24 | 27.63, 26.86, 25.01 | 24.52, 25.36, 22.72 | 13.21, 15.73, 13.66 | 17.31, 16.75, 14.53 | 15.18, 14.64, 15.48 | 10.53, 9.67, 10.10 | 11.67, 11.30, 8.53 | 10.58, 10.10, 7.83 | 6.32, 7.28, 4.40 |
| Flow conditions caused a significant reduction in macropinocytosis by infected macrophages (p>0.05 by one-way ANOVA*). Initial macrophage infection rate was >80% after 24 h, n=3.* | | | | | | | | | |
