# Supplementary material for: Comparative assessment of macrophage responses and antileishmanial efficacy in dynamic vs. Static culture systems utilizing chitosan-based formulations
Source: PLoS One. 2025 Mar 11;20(3):e0319610. doi: 10.1371/journal.pone.0319610 (PMC11896045; doi:10.1371/journal.pone.0319610)
Supplement: S13 Table — Values behind the means, standard deviations. (DOCX) [file pone.0319610.s013.docx]

**S13 Table: *In vitro* activity of chitosan solution and nanoparticles against *L. major* amastigotes in RPMI-1640 (pH=6.5) at different flow rates. Values behind the means, standard deviations.**

| Compound | Static-0 m/s | Flow-1.45 x 10⁻⁹ m/s | Flow-1.23 x 10⁻⁷ m/s |
| --- | --- | --- | --- |
| EC50 (µg/ml) | | | |
| Chitosan solution | 10.43, 12.29, 9.98 | 24.07, 22.34, 21.70 | 53.36, 58.05, 54.49 |
| Blank chitosan-TPP nanoparticles | 19.90, 10.24, 13.65 | 25.67, 33.02, 29.22 | 60.16, 50.46, 56.49 |
| AmB loaded chitosan-TPP nanoparticles | 0.10, 0.09, 0.11 | 0.39, 0.41, 0.41 | 1.08, 1.09, 1.13 |
| AmB solution (Pure) | 0.09, 0.08, 0.10 | 0.09, 0.11, 0.11 | 0.13, 0.09, 0.09 |
| EC90 (µg/ml) | | | |
| Chitosan solution | 159.39, 171.53, 164.08 | 210.63,232.19,247.18 | 443.42,465.37,456.21 |
| Blank chitosan-TPP nanoparticles | 276.92, 216.24, 229.84 | 346.8,263.96,286.25 | 372.44,541.29,463.28 |
| AmB loaded chitosan-TPP nanoparticles | 0.88, 1.13, 0.99 | 2.38,2.63,2.49 | 3.12,3.85,3.53 |
| AmB solution (Pure) | 0.49, 0.49, 0.53 | 0.83,1.04,0.83 | 1.59,1.55,1.36 |
