# Supplementary material for: Comparative assessment of macrophage responses and antileishmanial efficacy in dynamic vs. Static culture systems utilizing chitosan-based formulations
Source: PLoS One. 2025 Mar 11;20(3):e0319610. doi: 10.1371/journal.pone.0319610 (PMC11896045; doi:10.1371/journal.pone.0319610)
Supplement: S14 Table — major amastigotes infecting PEMs in pH = 6.5 under different flow conditions. (The data presented in this table were used to generate Fig 5, (A) and (B). (DOCX) [file pone.0319610.s014.docx]

| **S14 Table: Dose-response curve of the activity of chitosan solution (A), blank chitosan-TPP nanoparticles (B) against *L. major* amastigotes infecting PEMs in pH=6.5 under different flow conditions. (The data presented in this table were used to generate Figure 5, (A) and (B)** | | | | | | |
| --- | --- | --- | --- | --- | --- | --- |
|  | **Reduction in parasite infection%** | | | | | |
|  | **static system** | | **1.45 x 10^-9^ m/s** | | **1.23 x 10^-7^ m/s** | |
| **Concentration(µg/ml)** | **Chitosan solution** | **Blank chitosan-TPP nanoparticles** | **Chitosan solution** | **Blank chitosan-TPP nanoparticles** | **Chitosan solution** | **Blank chitosan-TPP nanoparticles** |
| 135 | 82,84,81 | 79,80,79 | 82,84,81 | 79,78,80 | 74,73,72 | 74,70,74 |
| 45 | 75,73,74 | 70,69,66 | 69,69,68 | 69,65,64 | 44,42,43 | 40,40,40 |
| 15 | 60,63,62 | 55,58,54 | 36,37,38 | 30,32,33 | 22,20,19 | 19,20,19 |
| 5 | 40,39,36 | 39,39,38 | 24,23,23 | 20,22,25 | 10,11,10 | 9,11,10 |
| 1.66 | 9,10,9 | 2,1,0 | 4,4,4 | 5,6,4 | 2,1,0 | 0,0,0 |
|  | | | | | | |
