# Supplementary material for: Comparative assessment of macrophage responses and antileishmanial efficacy in dynamic vs. Static culture systems utilizing chitosan-based formulations
Source: PLoS One. 2025 Mar 11;20(3):e0319610. doi: 10.1371/journal.pone.0319610 (PMC11896045; doi:10.1371/journal.pone.0319610)
Supplement: S15 Table — major amastigotes infecting PEMs in pH = 6.5 under different flow conditions. (The data presented in this table were used to generate Fig 5, (C) and (D). (DOCX) [file pone.0319610.s015.docx]

| **S15 Table: Dose-response curve of the activity of AmB loaded chitosan-TPP nanoparticles (C) and AmB solution (pure) (D) against *L. major* amastigotes infecting PEMs in pH=6.5 under different flow conditions. (The data presented in this table were used to generate Figure 5, (C) and (D)** | | | | | | |
| --- | --- | --- | --- | --- | --- | --- |
|  | **Reduction in parasite infection%** | | | | | |
|  | **static system** | | **1.45 x 10^-9^ m/s** | | **1.23 x 10^-7^ m/s** | |
| **Concentration(µg/ml)** | **AmB loaded chitosan-TPP nanoparticles** | **AmB solution (Pure)** | **AmB loaded chitosan-TPP nanoparticles** | **AmB solution (Pure)** | **AmB loaded chitosan-TPP nanoparticles** | **AmB solution (Pure)** |
| 2 | 96,98,96 | 100,100,100 | 96,98,96 | 85,87,84 | 78,77,74 | 80,81,79 |
| 0.6667 | 94,95,93 | 93.5,94.5,95.6 | 60,61,59 | 80,78,79 | 24,22,23 | 74,75,74 |
| 0.2222 | 55,50,49 | 70,74.5,73.9 | 32,34,33 | 70,73,72 | 12,11,10 | 69,68,71 |
| 0.0741 | 40,41,42 | 49.9,48.6,49.7 | 20,18,20 | 50,48,49 | 4,3,2, | 49,48,48 |
| 0.0247 | 12.5,11.9,12.3 | 14,13,14 | 4,5,4 | 10,9,10 | 0,0,0 | 8,7,6 |
| 0.0082 | 4,0,5 | 4,0,5 | 0,0,0 | 0,0,0 | 0,0,0 | 0,0,0 |
|  | | | | | | |
